# Proviral Quasispecies Diversity Is Not Associated With Virologic Breakthrough or CD4^+^ T Cell Loss in HIV-1 Elite Controllers

**DOI:** 10.3389/fmicb.2019.00673

**Published:** 2019-04-02

**Authors:** Suwellen S. D. de Azevedo, Fernanda H. Côrtes, Edson Delatorre, Marcelo Ribeiro-Alves, Brenda Hoagland, Beatriz Grinsztejn, Valdilea G. Veloso, Mariza G. Morgado, Gonzalo Bello

**Affiliations:** ^1^Laboratório de AIDS & Imunologia Molecular, Instituto Oswaldo Cruz, Fundação Oswaldo Cruz, Rio de Janeiro, Brazil; ^2^Laboratório de Pesquisa Clínica em DST e AIDS, Instituto Nacional de Infectologia Evandro Chagas, Fundação Oswaldo Cruz, Rio de Janeiro, Brazil

**Keywords:** elite controllers, HIV proviral diversity, plasma biomarkers, CD4^+^ T cell loss, breakthrough viremia

## Abstract

Elite controllers (EC) are able to control HIV-1 replication to extremely low levels (<50 HIV-1 RNA copies/mL) in the absence of antiretroviral therapy. However, some EC experience CD4^+^ T cell loss and/or lose their ability to control HIV-1 over the course of infection. High levels of HIV-1 *env* proviral diversity, activated T cells and proinflammatory cytokines were pointed out as relevant biomarkers for detection of EC at risk of virologic/immunologic progression. The aim of this study was to assess the importance of proviral diversity as a prognostic marker of virologic and/or immunologic progression in EC. To this end, we analyzed plasma viremia, total HIV DNA levels, T cells dynamics, and activation/inflammatory biomarkers in EC with low (EC_LD_ = 4) and high (EC_HD_ = 6) HIV-1 *env* diversity. None of EC_LD_ and EC_HD_ subjects displayed evidence of immunologic progression (decrease in absolute and percentage of CD4^+^ T cells) and only one EC_HD_ subject presented virologic progression (≥2 consecutive viral loads measurements above the detection limit) 2–5 years after determination of proviral *env* diversity. Despite differences in proviral genetic diversity, the EC_LD_ and EC_HD_ subgroups displayed comparable levels of total cell-associated HIV DNA, activated CD8^+^ T (CD38^+^HLA-DR^+^) cells and plasmatic inflammatory biomarkers (IP-10, IL-18, RANTES, PDGF-AA, and CTACK). These results indicate that the genetic diversity of the HIV-1 proviral reservoir is not a surrogate marker of residual viral replication, immune activation or inflammation, nor an accurate biomarker for the prediction of virologic breakthrough or CD4^+^ T cells loss in EC.

## Introduction

A rare subset of HIV-1–infected individuals, termed elite controllers (EC), is able to naturally suppress viral replication to levels <50 HIV-1 RNA copies/mL during chronic infection in absence of antiretroviral therapy (ART) (Deeks and Walker, 2007). Despite the extraordinary ability to naturally suppress HIV-1 viremia, a proportion of EC exhibit a CD4^+^ T cell counts decline (immunologic progression) and/or lose their ability to control HIV (virologic progression) over the course of infection (Andrade et al., 2008; Okulicz et al., 2009; Sajadi et al., 2009; Boufassa et al., 2011; Yang et al., 2012; Olson et al., 2014; Leon et al., 2016; Chereau et al., 2017). Identify those EC at risk of CD4^+^ T cell loss and/or of breakthrough viremia may help to guide the selection of individuals that may benefit from ART initiation.

Some EC subjects exhibit abnormally high levels of systemic T cell activation and inflammation that may contribute to both CD4^+^ T cell counts loss and breakthrough viremia (Andrade et al., 2008; Hunt et al., 2008; Noel et al., 2014, 2015b; Pernas et al., 2017). Virologic progression has been also associated with the increase of intermittent viremia episodes, total HIV DNA and HIV proviral diversity in the blood (Noel et al., 2015b; Pernas et al., 2017). High levels of CD8^+^ T cell activation and interferon gamma-induced protein 10 (IP-10) were pointed out as the most discriminant biomarkers for detection of EC at risk of immunologic progression (Noel et al., 2014, 2015b); while levels of HIV *env* diversity and plasma pro-inflammatory cytokines CCL5/RANTES (Pernas et al., 2017) and Galectin-3-binding protein (Rodríguez-Gallego et al., 2018) were described as useful baseline markers to predict virologic progression in EC 1 year before the loss of HIV control.

A recent study conducted by our group demonstrated two divergent patterns of intra-host proviral diversity in a cohort of Brazilian EC (de Azevedo et al., 2017). A subgroup of EC displayed highly homogeneous proviral quasispecies (mean *env* genetic diversity < 2%), consistent with the maintenance of the viral reservoir by clonal expansion of long-lived HIV-infected memory CD4^+^ T cells. The other subgroup of EC showed more diverse proviral populations (mean *env* genetic diversity ≥ 2%), consistent with residual evolution and continuous reseeding of the proviral reservoir. The precise association between these divergent patterns of intra-host *env* proviral diversity and the subsequent HIV-1 disease progression in our EC cohort was not addressed before.

Here, we evaluated the importance of HIV-1 *env* proviral diversity as a possible prognostic marker of immunologic and/or virologic progression in our EC cohort by analyzing the plasma viremia, total cell-associated HIV DNA levels, CD4^+^ T cells dynamics, CD8^+^ T cell activation and inflammatory biomarkers over infection course in EC harboring proviral quasispecies with low (EC_LD_ = 4) and high (EC_HD_ = 6) *env* diversity.

## Materials and Methods

### Study Subjects

The EC was defined as HIV-1–infected subjects with the most (≥70%) plasma viral load (VL) determinations under the detection limit (<50–80 copies/mL) in absence of ART as described previously (de Azevedo et al., 2017). Then, they were divided into two groups based on the proviral genetic diversity: (1) EC_LD_ for those harboring proviral quasispecies with a mean *env* genetic diversity < 2% (*n* = 4); and (2) EC_HD_ for those harboring proviral quasispecies with a mean *env* genetic diversity ≥ 2% (*n* = 6). All EC subjects were followed at least by 2 years after HIV-1 genetic diversity evaluation. A group of viremic controllers (VC) that displayed most (≥70%) VL determinations between 51 and 2,000 copies/mL (*n* = 8) was included as control. Immunologic progression was defined as a statistically significant decline in both absolute and percentage of CD4^+^ T cells (Burcham et al., 1991; Hulgan et al., 2007). Virologic progression in EC was defined as ≥ 2 consecutive detectable VL within 1 year (Pernas et al., 2017).

### CD4^+^ T Cell Counts, Plasmatic Viral Load and Total Cell-Associated HIV-1 DNA Load Measurements

CD4^+^ T cells counts and plasma HIV-1 RNA VL were determined as described previously (de Azevedo et al., 2017). Total DNA was extracted from PBMC (1 × 10^7^ cells) using the QIAamp DNA Mini Kit (Qiagen, Germany) and total cell-associated HIV-1 DNA load was quantified using the Generic HIV^®^DNA cell Kit (Biocentric, France), following the manufacturer’s recommendations. Results were reported either as actual numbers of HIV DNA copies/10^6^ cells or as the threshold value of detection.

### Analyses of Proviral Genetic Diversity

Proviral *env* sequences from PBMC were performed by single genome amplification (SGA) followed by analysis of viral diversity using conditions previously described (de Azevedo et al., 2017).

### Markers of T Cell Activation and Inflammation

To quantify the expression of CD38 and HLA-DR on CD8^+^ T cells, cryopreserved PBMC were thawed and stained with the following antibodies: anti-CD3 APC-H7, anti-CD4 PECF594, anti-CD8 APC, anti-CD38 BB515, and anti-HLA-DR PE (BD Biosciences, United States), and acquired using a BD FACSAria IIu Flow Cytometer (BD Biosciences, United States). The Fixable Viability Stain 450 (FVS 450-BD Biosciences, United States) was used to exclude non-viable cells. Flow cytometric analyses were performed with FlowJo v.10.0.7 (Tree Star Inc., Ashland, OR, United States). Plasmatic levels (pg/mL) of IP-10, IL-18, RANTES, CTACK, and PDGF-AA were measured using the human Magnetic Luminex Performance Assay (R&D systems, United States), following manufacturer’s instruction and the analyses were performed on a Luminex 200 System (Luminex, United States).

### Statistical Analysis

The Mann–Whitney *U*-test was used to compare data between EC_LD_ and EC_HD_ independent groups. The Wilcoxon signed-rank test was used to compare data between pre- and pos-diversity evaluation within either EC_LD_ or EC_HD_. Correlations between markers cumulative measures and HIV-1 DNA diversity were assessed by the Spearman correlation coefficient. The slopes of subgroups of T cells were calculated for each one by linear regression analysis fitted by generalized least squares and with an autocorrelation structure of first order in respect to the time after HIV diagnosis. Tests were two-sided, and *P* ≤ 0.05 were considered as significant. Graphics and statistical analyses were performed using either GraphPad v6 (Prism Software, United States) or R (R Foundation for Statistical Computing, Austria) software.

## Results

**FIGURE 1:**
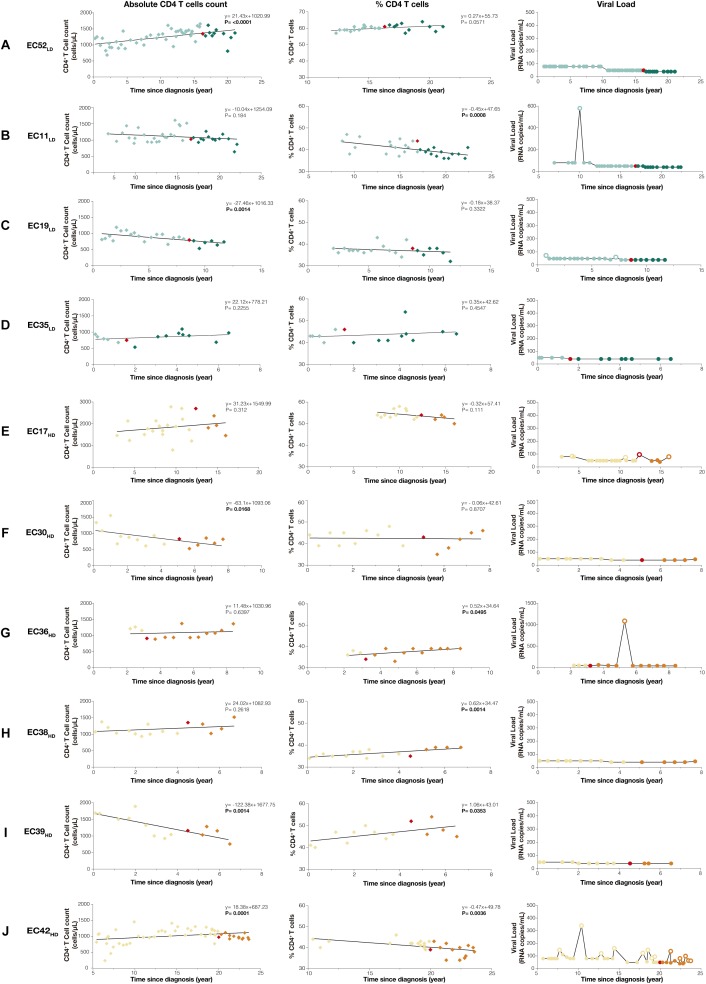
Temporal dynamics of CD4^+^ T cell counts and plasma viremia in EC_LD_ **(A–D)** and EC_HD_ **(E–J)** subgroups. Absolute CD4^+^ T cells counts (cells/μL), percentages of CD4^+^ T cell (%CD4) and plasma RNA viral load measurements (copies/mL) since HIV diagnosis are shown on the left, middle and right columns, respectively. The slope of CD4^+^ T cells and %CD4 overtime, calculated by linear regression analysis fitted by generalized least squares and with an autocorrelation structure of first order in respect to the time after HIV diagnosis, are shown with their respective *P-*values in the upper right corner of each graph. The red symbols (diamonds or circles) indicate the time point selected for analysis of *env* proviral diversity, while light and dark colored symbols indicate pre- and post-period of *env* proviral diversity determination, respectively. RNA viral load measurements below and above the detection limit were represented by fill and empty circles, respectively. EC_LD_ and EC_HD_ subjects were represented by green and orange symbols, respectively.

There were no statistically significant differences between EC_LD_ and EC_HD_ groups in terms of epidemiological (age, sex, and HIV-1 transmission), immunologic (absolute CD4^+^ and CD8^+^ T cells counts, %CD4 and CD4/CD8 ratio), genetic (HLA-B^∗^57/27 status) and virologic (plasma HIV-1 RNA, total HIV-1 DNA loads, and Hepatitis C status) characteristics at proviral diversity determination point or throughout the follow-up (Supplementary Table S1). Longitudinal analysis revealed no evidence of immunologic progression in our EC cohort (Figure 1). Subjects that showed a significant (*P* < 0.05) decrease in absolute CD4^+^ T cells counts over time (EC19_LD_, EC30_HD_, and EC39_HD_) maintained CD4^+^ T cells counts > 500 cells/mm3 and stable percentage of CD4^+^ T cells > 30%. Subjects that displayed significant (*P* < 0.01) decline in the percentage of CD4^+^ T cells (EC11_LD_ and EC42_HD_) maintained stable or increasing numbers of CD4^+^ T cells counts over time. All EC_LD_ and EC_HD_ subjects kept persistent virologic control for 2–5 years after determination of proviral *env* diversity, with exception of subject EC42_HD_ that displayed ≥2 consecutive VL measurements above the detection limit (59–97 copies/mL) in a 1-year period and thus lost the elite virologic control profile during follow-up (Figure 1 and Supplementary Table S2).

**FIGURE 2:**
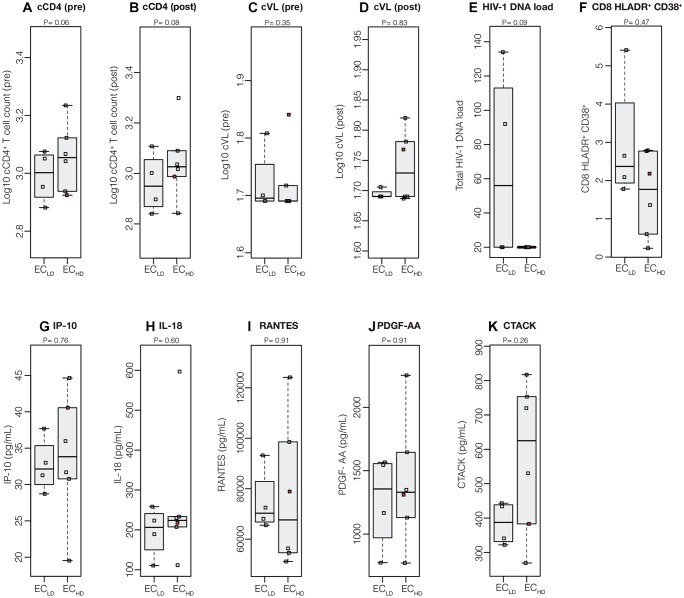
Virologic and immunologic progression markers in EC_LD_ and EC_HD_ subgroups. Mean (log_10_-transformed) cumulative measures of CD4^+^ T cell counts **(A,B)** and VL **(C,D)** in the pre- and post-periods respect to the point of determination of *env* proviral diversity and the total cell-associated HIV-1 DNA load **(E)**, frequency of activated CD8^+^ T cells **(F)**, and plasmatic levels of IP-10 **(G)**, IL-18 **(H)**, RANTES **(I)**, PDGF-AA **(J)**, and CTACK **(K)** at the *env* proviral diversity point were compared between EC subgroups. Central black solid bars and boxplots represent sample medians and interquartile, respectively. The red point in each graph indicate the values obtained for the subject who had putative virologic progression (EC42_HD_). *P*-values were obtained with Wilcoxon signed-rank tests.

Longitudinal analyses of the cumulative mean CD4^+^ T cell counts (cCD4^+^ T cell) and VL (cVL) in the pre- and post-periods of the proviral diversity assay date revealed no statistically significant differences between EC_LD_ and EC_HD_ subgroups (Figure 2A–D) nor within each subgroup between time periods (Supplementary Figure S1). Similarly, cross-sectional analyses of total cell-associated HIV DNA levels, cellular immune activation and plasma inflammatory biomarkers also did not distinguish both EC subgroups (Figure 2E–K). The EC42_HD_ subject, who had putative virologic progression after *env* diversity assay, presented most of the parameters within the range of other EC, except for cVL (pre) for which he exhibited the highest value of the group (Figure 2). The *env* proviral diversity was not significantly correlated with any of the virologic or immunologic parameter evaluated when EC were analyzed separately (Supplementary Figure S2). However, %CD8^+^CD38^+^HLA-DR^+^ T cells (*P* < 0.0001), total HIV DNA load (*P* = 0.003), and IP-10 (*P* = 0.03), were significantly higher in VC compared with EC (Supplementary Figure S3) and significant positive correlations with *env* proviral diversity (cVL, IP-10, and IL-18; Supplementary Figure S4A) and cVL (total HIV DNA load, %CD8^+^CD38^+^HLA-DR^+^ T cells, IP-10 and IL-18; Supplementary Figure S4B) were detected when HIV controllers (EC and VC) were taken as a whole.

## Discussion

In a previous study, we identified two subgroups of EC (EC_LD_ and EC_HD_) in a cohort of Brazilian subjects with divergent patterns of intra-host proviral diversity (de Azevedo et al., 2017). Our results comparing the clinical and epidemiologic aspects of the EC subgroups revealed no difference between them. Moreover, biomarkers previously associated with the risk of immunologic and/or virologic progression (CD4^+^ and CD8^+^ T cells counts, %CD4, CD8^+^ T cell activation, inflammation, HIV-1 RNA and DNA loads) in EC were not significantly different either before or after the proviral diversity assay among EC subsets.

The combined use of absolute and percentage CD4^+^ T cells trends over time reinforce the absence of true immunologic damage in our EC cohort. Subjects with a decrease in absolute CD4^+^ T cell counts showed stable (EC19_LD_ and EC30_HD_) or increasing (EC39_HD_) %CD4^+^ T cells, indicating that reduction was not specific to CD4^+^ T cells (Supplementary Figure S5). Subjects with a significant decrease in the %CD4^+^ T cells displayed stable (EC11_LD_) or even increasing (EC42_HD_) absolute CD4^+^ T cells counts. Furthermore, all EC_LD_ and EC_HD_ subjects maintained CD4^+^ T cell counts > 500 cells/mm^3^ and percentage of CD4^+^ T cells > 30% during follow-up, supporting a low risk for disease progression (Burcham et al., 1991; Hulgan et al., 2007). The only subject with a virologic breakthrough (EC42_HD_) maintained VL in the very low range (59–97 copies/mL) and it could be argued that he did not display a true virologic progression (>2,000 copies/mL) (Noel et al., 2015b; Chereau et al., 2017). These data show that immunologic and/or virologic progression was rare in both EC_LD_ and EC_HD_ groups and reinforce the need to unify criteria for the definition of true progression in EC.

A recent study described higher levels of HIV *env* diversity and proinflammatory cytokines (RANTES, PDGF-AA, and CTACK) in EC that lost virologic control 1 year later, compared with EC that maintained persistent virologic control (Pernas et al., 2017). Our analyses did not reveal significant differences in the levels of RANTES, PDGF-AA, and CTACK between EC_LD_ and EC_HD_ subgroups. Similarly, levels of CD8^+^ T cell activation and IP-10, previously pointed out as biomarkers for detection of EC at risk of immunologic progression (Noel et al., 2014; Côrtes et al., 2018), were also not significantly different between the EC_LD_ and EC_HD_ groups. Noteworthy, the only EC subject who had allegedly virologic progression in our cohort (EC42_HD_) displayed similar levels of immune activation and inflammatory biomarkers than subjects with persistent elite virologic control.

These results support that some EC are able to maintain stable CD4^+^ T cells and persistent control of viral replication for several years (>2–5 years) in the setting of high proviral diversity. Although HIV-1 diversity and other surrogate markers of viral replication (total HIV-1 DNA load, CD8^+^ T cell activation, and IP-10) were positively correlated among each other and with the cVL when EC and VC were taken as a whole, consistent with previous findings (Bello et al., 2005; Sajadi et al., 2007; Groves et al., 2012; Côrtes et al., 2015; Noel et al., 2015a; Platten et al., 2016; Canouï et al., 2017; Tarancon-Diez et al., 2018); we did not detect any significant association between the *env* proviral diversity and those biomarkers when EC were analyzed separately. Hence, the genetic diversity of the HIV-1 proviral reservoir in many EC is probably not driven by continuous residual viral replication (de Azevedo et al., 2017), which may explain why EC_HD_ does not seem to display higher risk of immunologic or virologic progression in compared with EC_LD_ in our cohort. Those EC for which diversity of HIV-1 proviral reservoir truly reflects continuous viral replication and persistent inflammation are probably the only ones at risk of immunologic or virologic progression.

The main limitation of this study was the small number of EC individuals analyzed, which may have resulted in the apparent absence of significant differences in various markers evaluated between EC subgroups. This limitation was partially counterbalanced by the well recorded long period of follow-up of these extremely rare group of patients and the very rigorous classification criteria used to ensure that only EC individuals with a long-lasting HIV-1 control profile were included in our cohort (Walker and Yu, 2013). Further studies comprising a larger number of individuals are needed to complement our findings and to define the most suitable combination of biomarkers necessary to predict immunologic and/or virologic progression in long-term EC.

## Conclusion

Our data suggest that genetic diversity of the HIV-1 proviral reservoir is not a surrogate marker of residual viral replication, immune activation or inflammation, nor an accurate biomarker for the prediction of virologic breakthrough or CD4^+^ T cells loss in EC. Most EC in our cohort maintained a persistent control of viremia and stable CD4^+^ T cells for up to 5 years after determination of HIV-1 quasispecies composition, irrespective of proviral genetic diversity. Understand the mechanisms leading to the divergent patterns of intra-host viral diversity in EC is of paramount importance to determine the potential impact of such divergent patterns on the long-term natural control of HIV-1 infection and their relevance for clinical management of EC.

## Data Availability

All datasets generated for this study are included in the manuscript and/or the Supplementary Files.

## Ethics Statement

All participants provided written informed consent and the ethical committee of Instituto Nacional de Infectologia Evandro Chagas (INI-Fiocruz) approved the study (CAAE 1717.0.000.009-07).

## Author Contributions

GB conceived and designed the study and supervised the experiments. SA and FC conducted the experiments and analyzed the data. ED performed the quantification of total cell-associated HIV-1 DNA, its analysis, and provided intellectual input. MR-A analyzed the data. BH, BG, and VV conducted the patient recruitment and follow-up. MM contributed to the study design and provided intellectual input. SA, FC, and GB wrote the first draft. All authors assisted with the writing and approved the final manuscript.

## Conflict of Interest Statement

The authors declare that the research was conducted in the absence of any commercial or financial relationships that could be construed as a potential conflict of interest.
