# Supplementary material for: Proviral Quasispecies Diversity Is Not Associated With Virologic Breakthrough or CD4+ T Cell Loss in HIV-1 Elite Controllers
Source: Front Microbiol. 2019 Apr 2;10:673. doi: 10.3389/fmicb.2019.00673 (PMC6454058; doi:10.3389/fmicb.2019.00673)
Supplement: Supplementary file 1 [file Data_Sheet_1.PDF]

Supplementary Material

Proviral quasispecies diversity is not associated with virologic breakthrough or $CD4^+$ T cell loss in HIV-1 elite controllers

Suwellen. S. D. de Azevedo[1]*, Fernanda H. Côrtes[1]*, Edson Delatorre[1], Marcelo Ribeiro-Alves[2], Brenda Hoagland[2], Beatriz Grinsztejn[2], Valdilea G. Veloso[2], Mariza G. Morgado[1] and Gonzalo Bello[1**]

***** These authors contributed equally.

****Correspondence:**

Gonzalo Bello

gbello@ioc.fiocruz.br / gbellobr@gmail.com

Supplementary Figures

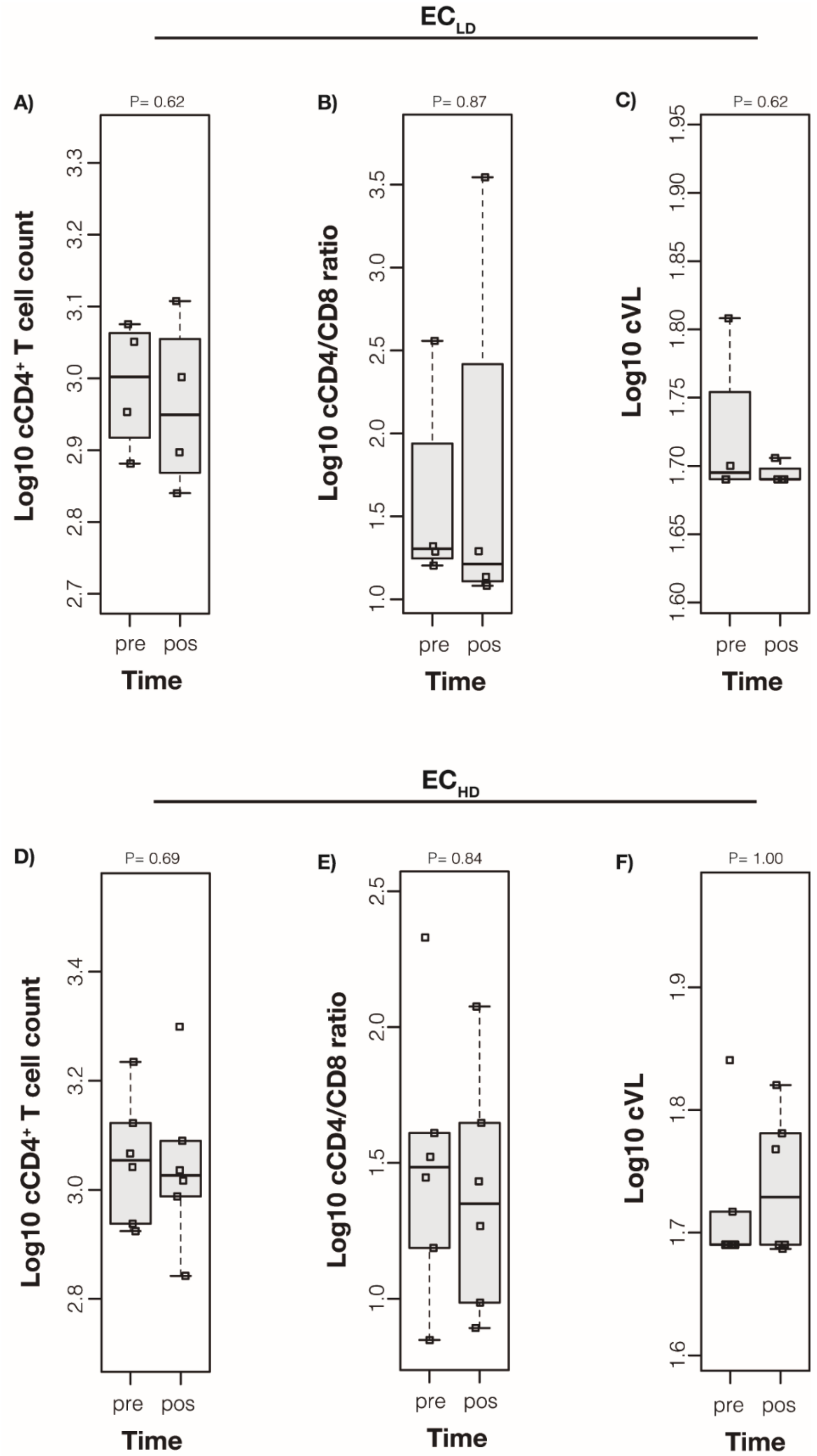

Supplementary Figure S1. Immunologic and virologic characteristics in pre- and post-period of determination of *env* diversity in EC_{LD} and EC_{HD}. Cumulative measurements (log_{10} – transformed) of the $CD4^+$ T cell count (A, D), CD4/CD8 ratio (B, E) and viral load (C, F) between pre- and post-period of determination of *env* diversity in EC subgroups. Central black solid bars and boxplots represent sample medians and interquartile, respectively. *P*-values were obtained with Wilcoxon signed rank tests.

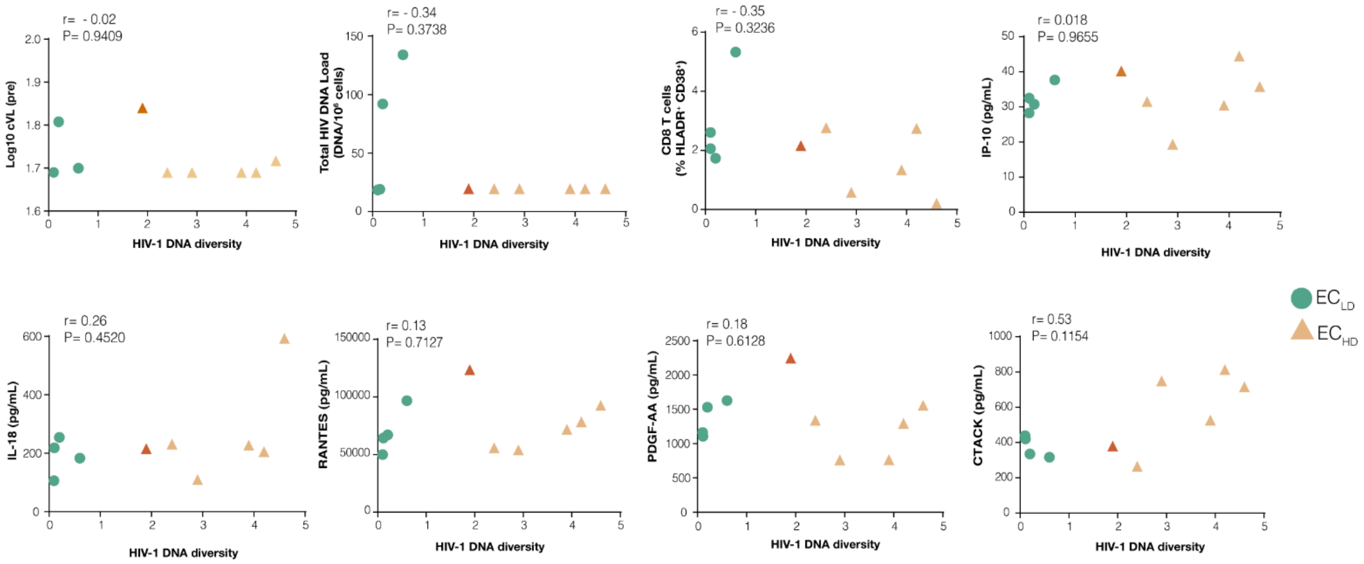

Supplementary Figure S2. Relationships between *env* proviral diversity and virologic/immunologic parameters in EC. Spearman's Rank Correlations Coefficient analysis was performed between *env* proviral diversity and cumulative measurements of the viral load (cVL, log_{10} – transformed), total HIV-1 DNA load, proportion of HLA-DR^+CD38^+ $CD8^+$ T cells, and levels of the IP-10, IL-18, RANTES, PDGF-AA and CTACK. The coefficient of correlation (r) and their respective *P*-values are shown in the upper left corner of each graph. The green circles and light orange triangles in each graph represent the EC_{LD} and EC_{HD} subjects, respectively. The dark orange triangles in each graph represent the subject with putative loss of virologic control ($EC42_{HD}$).

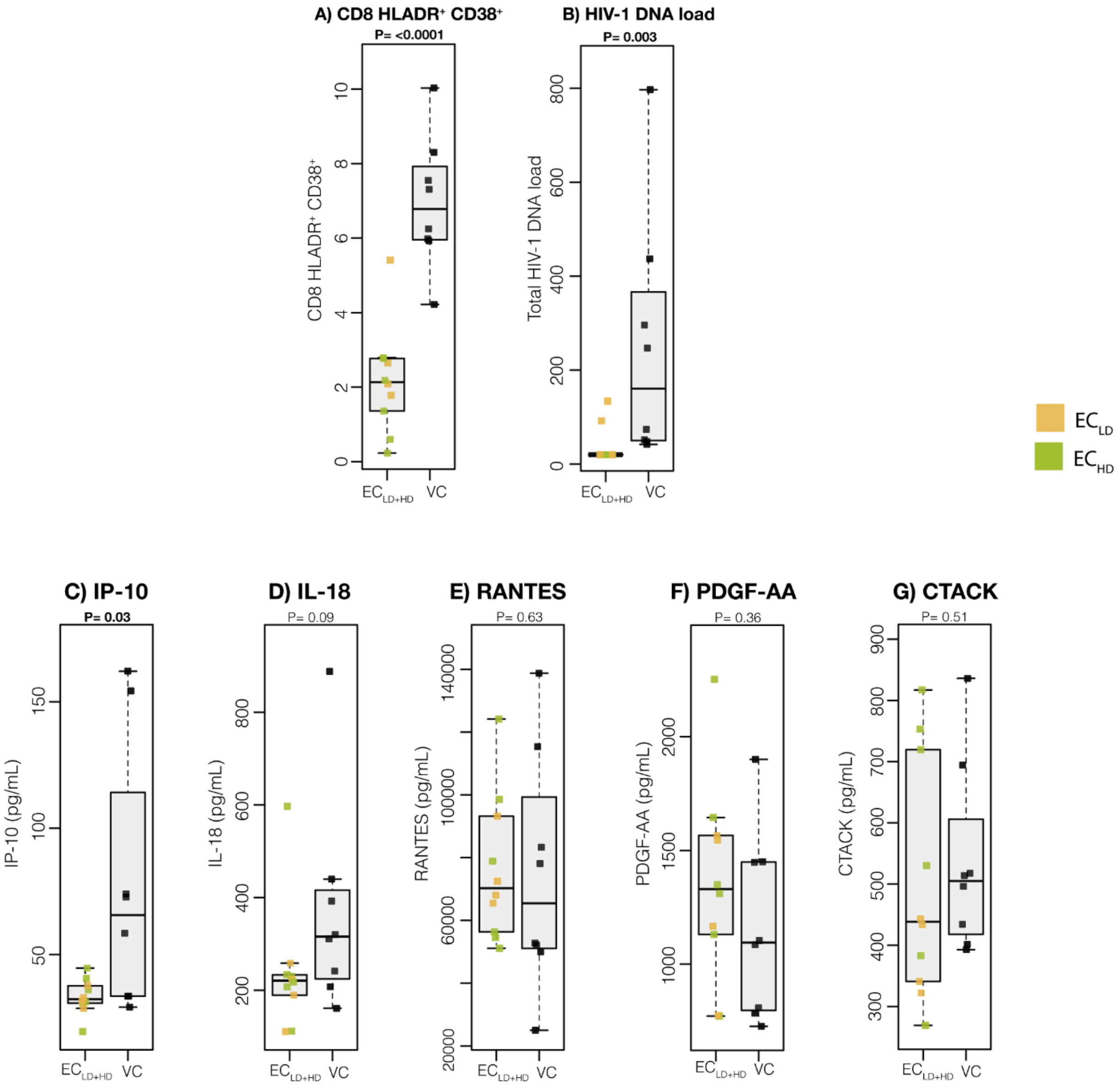

Supplementary Figure S3. Comparison of virologic and immunologic characteristics between the EC and VC. $CD8^{+}$ T cell activation (A), total HIV-1 DNA load (B) and plasmatic markers of inflammation/immune activation (C-H). The orange and green points in each graph indicate the EC_{LD} and EC_{HD} subjects, respectively. Central black solid bars and boxplots represent sample medians and interquartile, respectively. *P*-values were obtained with Mann–Whitney U tests.

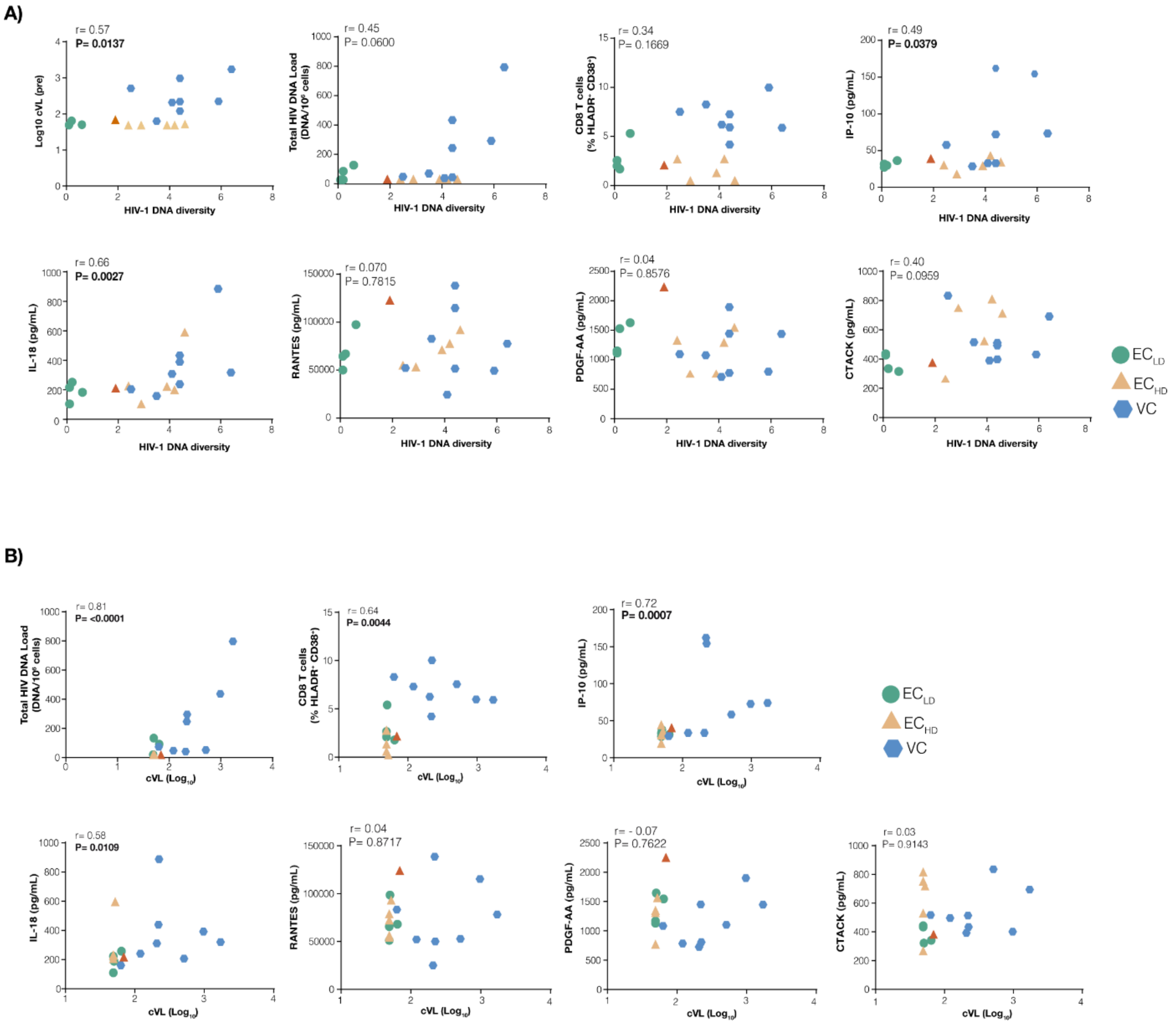

Supplementary Figure S4. Relationships between *env* proviral diversity (A) or cVL (B) with virologic/immunologic parameters in HIV controllers (EC and VC). Spearman's Rank Correlations Coefficient analysis was performed between *env* proviral diversity and cVL, total HIV-1 DNA load, proportion of HLA-$DR^{+}CD38^{+}$ $CD8^{+}$ T cells, and levels of the IP-10, IL-18, RANTES, PDGF-AA and CTACK. The coefficient of correlation (r) and their respective *P*-values are shown in the upper left corner of each graph. The green circles and light orange triangles in each graph represent the EC_{LD} and EC_{HD} subjects, respectively. The dark orange triangles in each graph represent the subject with putative loss of virologic control ($EC42_{HD}$).

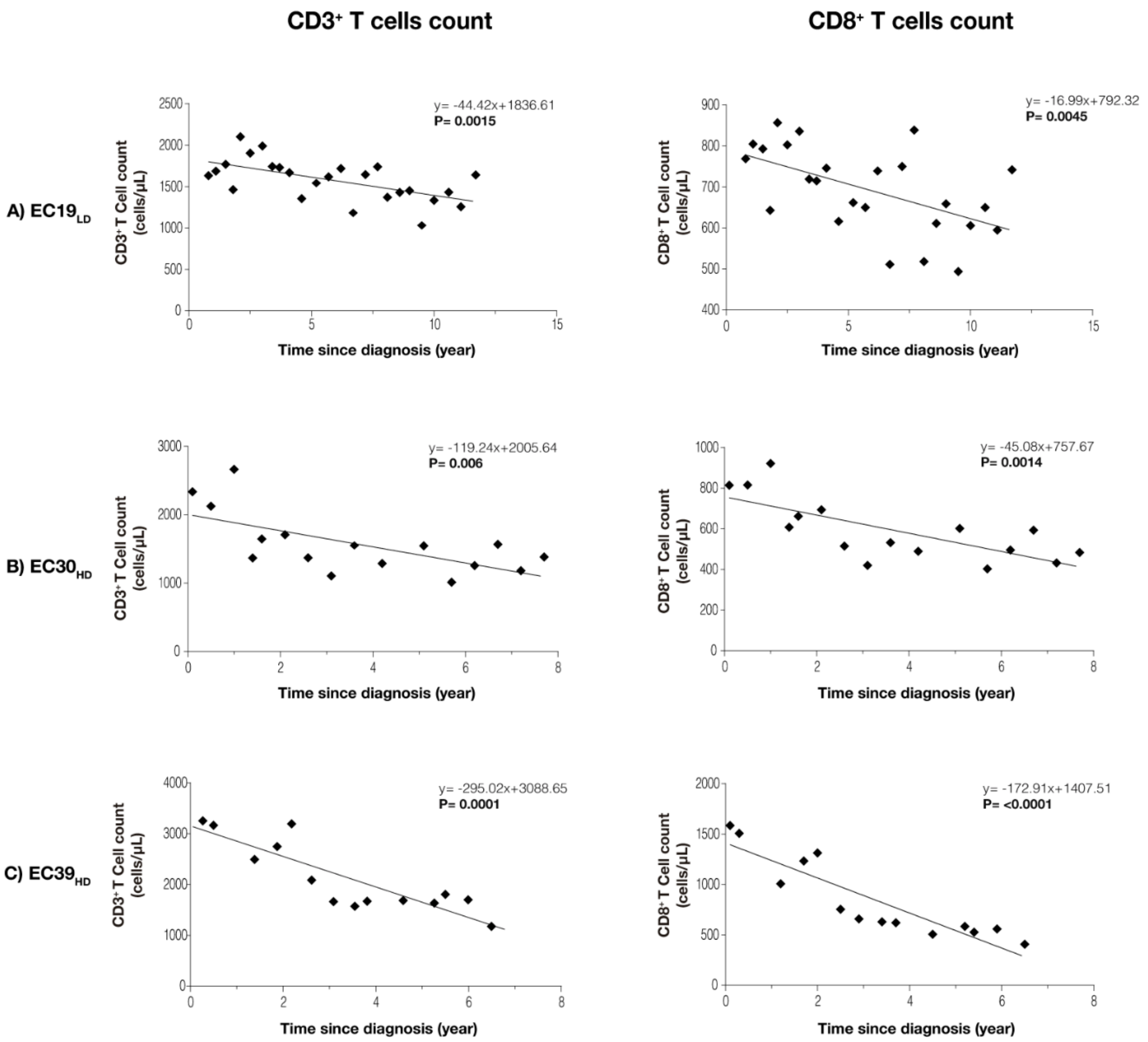

Supplementary Figure S5. $CD3^+$ and $CD8^+$ T cell dynamics in selected EC subjects. Slopes of absolute $CD3^+$ and $CD8^+$ T cells counts since HIV diagnosis are shown on the Y-axis of left and right columns, respectively. The slopes and their respective *P*-values are shown in the upper right corner of each graph and were calculated by linear regression analysis fitted by generalized least squares and with an autocorrelation structure of first order in respect to the time after HIV diagnosis.

Supplementary Tables

Supplementary Table S1. Clinical and epidemiologic characteristics EC_{LD} and EC_{HD} subgroups.

Parameter	EC_{LD} (n=4)	EC_{HD} (n=6)	*P* value*
Age (years)**	43.5 (35.0 – 46.5)	48.5 (35.5 – 65.0)	0.70
Sex (female), no. (%)	3 (75%)	5 (83%)	> 0.99
Heterosexual transmission, no. (%)	4 (100%)	5 (83%)***	0.67
HLA-B*57/27 presence, no. (%)	2 (50%)	1 (17%)	0.50
Hepatitis C coinfection, no. (%)	0	1 (17%)	> 0.99
**Study point**			
Time since of HIV-1 diagnosis (year)	12.4 (3.4 – 16.7)	4.8 (4.2 – 14.0)	0.72
$CD4^+$ T cell count (cells/mm^3)	912 (763 – 1267)	1048 (891 – 1688)	0.33
% CD4	45.0 (39.5 – 57.2)	41.0 (34.8 – 52.5)	0.47
$CD8^+$ T cell count (cells/mm^3)	544 (432 – 742)	856 (578 – 1597)	0.11
CD4/CD8 ratio	1.44 (1.3 – 2.8)	1.24 (0.98 – 1.87)	0.47
Plasma HIV RNA load (copies/ml)	49 (12.25 – 49)	49 (49 – 60.75)	0.53
Total HIV DNA load (DNA/10^6 cells)	56 (20 – 123)	20 (20 – 20)	0.13
**Follow-up**			
Time maintaining EC status (years)	16 (8.18 – 21.85)	8 (6.65 – 17.90)	0.47
$CD4^+$ T cell count (cells/mm^3)	981 (845 – 1215)	1123 (929 – 1424)	0.33
% CD4	43.0 (39.0 – 56.0)	41.5 (35.5 – 48.0)	0.52
$CD8^+$ T cell count (cells/mm^3)	682 (519 – 889)	851 (724 – 1006)	0.33
CD4/CD8 ratio	1.25 (1.2 – 2.4)	1.4 (1.2 – 1.8)	0.63
$CD4^+$ T cell slope (cells/mm^3/year)	5.5 (-23.08 – 22.08)	11.90 (-77 – 26.15)	> 0.99
%CD4 slope	0.34 (-0.45 – 0.35)	0.22 (-0.40-0.72)	0.48
cVL (log10–transformed)	1.7 (1.7-1.8)	1.7 (1.7-1.7)	0.92

* Statistical analyses were performed using the Mann–Whitney test. **Age at study point. ***A female subject had unknown HIV mode of transmission. cVL: cumulative viral load.

Supplementary Table S2. Virologic characteristics of EC_{LD} and EC_{HD} subgroups.

**Patient**	**Subtype**	**HIV DNA π**	**HIV DNA load (cp/10^6 cells)**	**VL Blip Frequency**	**VL Blip Amplitude (cp/mL)**	**Virologic breakthrough**
EC_{LD}52	B	0.1%	20	0/10	-	No
EC_{LD}11	B	0.2%	92	0/14	-	No
EC_{LD}19	B	0.6%	134	0/5	-	No
EC_{LD}35	F1	0.1%	20	0/9	-	No
**Mean**	**-**	**0.3%**	**67**	**0/38**	**-**	**0/4**
EC_{HD}17	B	4.6%	20	2/4	51-96	No
EC_{HD}30	B	2.9%	20	0/6	-	No
EC_{HD}36	B	2.4%	20	2/10	61-1,086	No
EC_{HD}38	A	3.9%	20	0/5	-	No
EC_{HD}39	B	4.2%	20	0/5	-	No
EC_{HD}42	B	1.9%	20	6/13	59-97	Yes
**Mean**	**-**	**3.3%**	**20**	**10/50**	**-**	**1/6**

VL: viral load.
